# Association between isolated abnormal 1-hour glucose challenge test and adverse pregnancy outcomes: a retrospective review from an urban tertiary care center in the United States

**DOI:** 10.1186/s12884-025-07214-x

**Published:** 2025-02-11

**Authors:** Hillary Hosier, Lisbet S. Lundsberg, Jennifer Culhane, Caitlin Partridge, Moeun Son

**Affiliations:** 1 Department of Obstetrics, Gynecology and Reproductive Sciences, Yale School of Medicine, 333 Cedar St, New Haven, CT 06510 USA; 2 Department of Maternal-Fetal Medicine, Weill-Cornell Medicine, New York, NY 10021 USA

**Keywords:** Glucose challenge, Glucose tolerance, Glucose intolerance, Glucose dysregulation, Gestational diabetes, Perinatal outcomes

## Abstract

**Background:**

The objective of this study was to investigate whether an isolated abnormal 1-hour glucose challenge test (GCT) among patients without gestational diabetes (GDM) is associated with adverse outcomes.

**Methods:**

This is a retrospective cohort study of patients who underwent GDM screening at ≥ 24 weeks’ gestation with a 1-hour GCT and delivered a singleton fetus at > 35 weeks’ gestation at an urban tertiary hospital from 1/2013 to 10/2021. Data were extracted from an electronic medical record data warehouse using standardized billing/diagnosis codes. Individuals were categorized into 3 groups: normal screening (1-hour GCT value < 140 mg/dL), intermediate screening (1-hour GCT value ≥ 140 and < 200 but normal 3-hour glucose tolerance test (GTT)), and GDM (1-hour GCT ≥ 200 mg/dL or abnormal 3-hour GTT). The primary composite perinatal morbidity outcome included any of the following: large for gestational age (LGA) birthweight, birth injury, hypoglycemia with neonatal intensive care unit (NICU) admission, respiratory distress syndrome, transient tachypnea of the newborn, apnea, NICU admission, or perinatal death. Multiple secondary outcomes were also evaluated. Bivariable analyses and multivariable logistic regression modeling were performed.

**Results:**

Of 37,277 eligible patients, 29,698 (79.7%) had normal screening results, 5092 (13.7%) had intermediate screening results, and 2487 (6.6%) were diagnosed with GDM. There were significant differences in baseline characteristics between the three groups, including age, parity, race and ethnicity, payer-type, obesity, and pre-pregnancy metformin use. Compared to normal screening, intermediate screening was associated with an increased risk for the composite perinatal morbidity outcome (OR 1.23, 95% CI 1.15–1.32), cesarean (OR 1.37, 95% CI 1.28–1.46), and hypertensive disorders of pregnancy (OR 1.30, 95% CI 1.20–1.40). Associations for these outcomes were further pronounced in those with GDM compared to normal screening (OR 1.86, 95% CI 1.70–2.03; OR 1.69, 95% CI 1.56–1.84; and OR 1.57, 95% CI 1.42–1.74, respectively). After adjusting for potential confounders, increased risks for the composite perinatal morbidity outcome persisted for those with intermediate screening (aOR 1.18, 95% CI 1.10–1.26).

**Conclusions:**

In addition to patients with GDM, individuals an isolated abnormal 1-hour GCT without GDM were also at increased risks for adverse pregnancy outcomes. Further investigation is needed to understand if patients with mild dysregulation may still benefit from other interventions.

**Supplementary Information:**

The online version contains supplementary material available at 10.1186/s12884-025-07214-x.

## Background

Normal pregnancy is a state of mild physiologic insulin resistance and about 8% of pregnancies are affected by gestational diabetes mellitus (GDM), a pathologic form associated with increased risks of maternal and perinatal complications [1–3]. In the United States, GDM screening is commonly performed using the 2-step method with an initial 1-hour glucose challenge test (GCT) using a 50-gram glucose load at 24–28 weeks of gestation, and if elevated, followed by a 3-hour glucose tolerance test (GTT) using a 100-gram glucose load [2]. Approximately 15–20% of patients who undergo the 1-hour GCT have elevated values, requiring the second step with the 3-hour GTT [4]. If GDM is diagnosed, life-style changes, nutritional counseling, and anti-diabetogenic medications are employed because improving maternal hyperglycemia has been shown to improve pregnancy outcomes [5, 6]. However, it remains unclear whether patients who have an abnormal initial GCT but normal second step (3-hour GTT) result, therefore avoiding a diagnosis of GDM, still have a degree of increased glucose intolerance that is associated with adverse pregnancy outcomes.

Therefore, we sought to examine individuals with an isolated abnormal GCT value but normal GTT test (herein described as intermediate screening results), with the hypothesis that those with intermediate screening results are at increased risk for adverse pregnancy outcomes compared to those with normal screening. Additionally, we hypothesized that there would be a dose-response relationship, with the greatest risk of adverse perinatal outcomes occurring in the group of individuals diagnosed with GDM.

## Methods

We performed a retrospective cohort study of all patients with a documented late second or third trimester 1-hour GCT result delivering at a single urban tertiary hospital from January 30, 2013 to October 2, 2021. Individuals were considered to have undergone a late second or third trimester GCT if the result occurred at ≥ 24 weeks 0 days of gestation using the estimated date of delivery (EDD) extracted from the delivery summary. Actual laboratory values were directly extracted from the medical records. At our institution, the two-step method is used starting with a 1-hour GCT using a 50-gram glucose load followed by a 1-hour venous blood draw. If the result is ≥ 140 mg/dL, a fasting 3-hour GTT with a 100-gram glucose load is recommended. Patients with a GCT result ≥ 200 mg/dL are presumptively diagnosed as having GDM [4] and not recommended to undergo 3-hour GTT testing. GDM is diagnosed with ≥ 2 elevated values on the GTT using the Carpenter and Coustan criteria [7] in accordance with the American Diabetes Association recommendations [8] with the following venous glucose level thresholds: fasting > 95 mg/dL, 1-hour > 180 mg/dL, 2-hour > 155 mg/dL, 3-hour > 140 mg/dL. Patients with a diagnosis of pre-gestational diabetes, multifetal gestation, and those delivered < 35 weeks of gestation were excluded from this study. Patients without gestational diabetes and without glucose challenge test ≥24 weeks of gestation were further excluded from the sample. The gestational age threshold was chosen because it is institutional policy that all newborns born < 35 weeks of gestation are automatically admitted to the NICU, a component of the composite outcome.

The exposure of interest was the GCT screening result. All five test values were reviewed for each patient to identify those with an abnormal result. Eligible patients were categorized into three study groups: normal screening result (1-hour GTT value < 140 mg/dL), intermediate screening result (1-hour GCT ≥ 140 mg/dL but < 200 mg/dL and normal 3-hour GTT (< 2 values elevated)), and GDM (1-hour GCT ≥ 200 or abnormal 3-hour GTT with ≥ 2 values elevated).

The primary study outcome was a composite of perinatal morbidity and mortality which included any of the following for the infants: large for gestational age (LGA) birthweight, birth injury, neonatal hypoglycemia with neonatal intensive care unit (NICU) admission, respiratory distress, NICU admission, or stillbirth. Secondary outcomes included cesarean delivery, obstetric anal sphincter injury (OASIS), hypertensive disorders of pregnancy (HDP), and small for gestational age (SGA) birth weight. LGA and SGA were defined as birthweights > 90th percentile and < 10th percentile, respectively, using sex-specific nomograms by Aris et al. [9] NICU admission and stillbirth were extracted directly from the electronic medical record (EMR) based on standardized templated fields. All other variables were obtained using International Classification of Diseases (ICD) codes (Supplemental Table S1). Birth injury included brachial plexus injury, Erb’s palsy, Klumpke’s palsy, or fracture of the clavicle, femur, humerus, skull or other fracture. Respiratory distress included meconium aspiration syndrome, neonatal bronchopulmonary disorder, and other neonatal respiratory distress. HDP included gestational hypertension, pre-eclampsia, eclampsia, or hemolysis, elevated liver enzymes and low platelet count syndrome.

Baseline demographic data were collected from the EMR including maternal age, parity, race and ethnicity, marital status, payer type, pre-pregnancy body mass index (BMI), smoking status, and gestational age at GDM screening. At our institution, race and ethnicity are typically recorded as self-reported by the patient and documented in the EMR. Marital status is categorized as either married or living as married, or single, divorced, widowed, or other/unknown. Baseline medical comorbidities including chronic hypertension, chronic kidney disease, and conception via in vitro fertilization (IVF) were collected based on ICD codes (Supplemental Table S1). The use of certain medications in pregnancy were also collected. Metformin use prior to pregnancy was examined because it is a biguanide that inhibits hepatic gluconeogenesis and glucose absorption and stimulates glucose uptake in peripheral tissues [10]. The administration of antenatal steroids (e.g., betamethasone) was also examined as a potential confounder for the primary outcome [11]. Additionally, given the known benefit of low dose aspirin to prevent preeclampsia [12], data were collected on maternal aspirin use in pregnancy. Gestational age at delivery, mode of delivery (vaginal vs. operative vs. cesarean delivery), and fetal sex were also collected. We evaluated predictors for neonatal outcomes based on variables utilized by the National Institute of Child Health and Development extremely preterm birth outcomes tool including fetal sex, gestational age, birth weight, and receipt of antenatal steroids (https://www.nichd.nih.gov/research/supported/EPBO/use).

We performed bivariate analyses using chi-square tests or Fischer’s exact tests, as appropriate, for categorical variables and Kruskal-Wallis tests for continuous variables. We examined the association between our exposure variable and the composite of perinatal morbidity and then adjusted for potential variables using multivariable logistic regression models. We adjusted for potential covariates using the maternal and fetal characteristics with p-values < 0.05 in bivariate analyses. The normal screening group was used as the referent group for all comparisons. Odds ratios (ORs) and 95% confidence intervals (CIs) were calculated. All analyses were performed using SA 9.4 (SAS Institute, Cary, NC). This study had Institutional Review Board approval with a waiver for informed consent (IRB number 1605017853, initial approval date 6/24/20216).

## Results

**Fig. 1:**
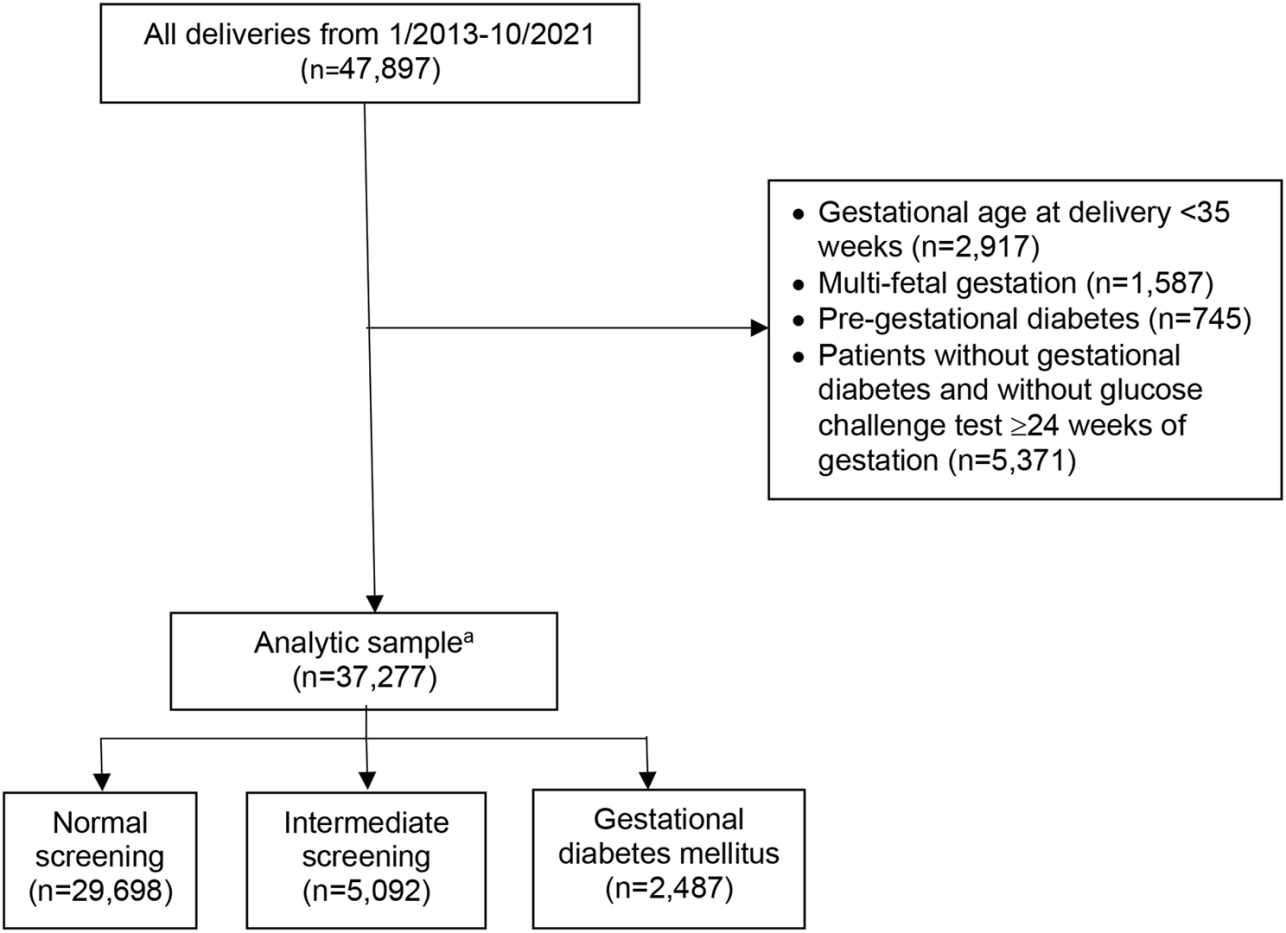
Flow diagram to define study cohort, *N* = 37,277.^**a**^Eligible patients were categorized into three study groups: normal screening (1-hour GTT value < 140 mg/dL), intermediate screening or isolated abnormal GCT screening (1-hour GCT ≥140 mg/dL but < 200 mg/dL and normal 3-hour GTT (< 2 values elevated)), and gestational diabetes mellitus (1-hour GCT > 200 or abnormal 3-hour GTT with 2 or more values elevated)

Of the 47,897 deliveries during the study period, 37,277 (78%) were eligible for analysis. Of the study sample, 29,698 (79.7%) had normal screening results, 5,092 (13.7%) had intermediate screening results, and 2,487 (6.6%) were diagnosed with GDM (Fig. 1). There were significant differences in the distributions of maternal age, parity, race and ethnicity, insurance type, BMI, marital status, and medical comorbidities between groups (Table 1). Notably, the percentage of patients with BMI > 30 kg/m^2^ in the GDM group was 46.4%, which is significantly higher than in the intermediate group (30.8%) or normal screening group (24.5%, *p* < 0.0001). Aspirin use increased across the screening categories (5.7% normal, 7.7% intermediate, and 15.6% GDM; *p* < 0.001) as did preconception Metformin use (0.8% normal, 1.3% intermediate, and 3.0% GDM, *p* < 0.0001). There were no significant differences in Betamethasone use during delivery hospitalization (Table 2). There was a significant difference in gestational age at delivery across the cohorts, though this difference is not clinically significant as the median range for all cohorts was within the 39th week (Table 2).

**Table 1:** Baseline characteristics by glucose screening result grouping

Characteristic	Normal screening (*n* = 29,698)	Intermediate screening (*n* = 5,092)	Gestational diabetes mellitus (*n* = 2,487)	*p*-value
Age at delivery, years	30.0 (26.0–34.0)	32.0 (29.0–35.0)	33.0 (29.0–36.0)	< 0.0001
Married or partnered	18,609 (62.7)	3531 (69.3)	1654 (66.5)	< 0.0001
Race and Ethnicity Hispanic Black, Non-Hispanic White, Non-Hispanic Asian, Non-Hispanic Other, Non-Hispanic	6212 (20.9) 5240 (17.6) 15,656 (52.7) 1537 (5.2) 1053 (3.6)	1041 (20.4) 604 (11.9) 2801 (55.0) 421 (8.3) 225 (4.4)	584 (23.5) 378 (15.2) 1101 (44.3) 299 (12.0) 125 (5.0)	< 0.0001
Nulliparous^a^	12,727 (43.6)	2121 (42.3)	977 (39.9)	0.0009
Commercial insurance	17,543 (59.1)	3335 (65.5)	1450 (58.3)	< 0.0001
Pre-pregnancy BMI ≥ 30 kg/m^2 b^	6232 (24.5)	1381 (30.8)	1047 (46.4)	< 0.0001
Active smoker or quit in pregnancy	2198 (7.4)	363 (7.1)	158 (6.4)	0.1376
Preconception metformin use	226 (0.8)	66 (1.3)	75 (3.0)	< 0.0001
Chronic hypertension	1933 (6.5)	406 (8.0)	296 (11.9)	< 0.0001
Chronic kidney disease	238 (0.8)	35 (0.7)	15 (0.6)	0.4199
In-vitro fertilization	1087 (3.7)	282 (5.5)	150 (6.0)	< 0.0001
Low dose aspirin use	1693 (5.7)	392 (7.7)	387 (15.6)	< 0.0001

Data are presented as median (interquartile range) and n (%)

Bivariate analyses performed using chi-square tests or Fischer’s exact tests for categorical values and Kruskal-Wallis tests for continuous variables

GDM = gestational diabetes mellitus; GCT = glucose challenge test; GTT = glucose tolerance test; BMI = body mass index;

^a^Missing values for 491 (1.7%) in the normal screening group, 78 (1.5%) in the isolated abnormal 1-hour GCT group, and 40 (1.6%) in the GDM group

^b^Missing values for 4205 (14.1%) in the normal screening group, 610 (12.0%) in the isolated abnormal 1-hour GCT group, and 232 (9.3%) in the GDM group

**Table 2:** Fetal characteristics by glucose screening result grouping

	Normal screening (*n* = 29,698)	Intermediate screening (*n* = 5,092)	Gestational diabetes mellitus (*n* = 2,487)	*P* value
Male fetal sex	15,139 (51.0)	2568 (50.4)	1242 (49.9)	0.1626
Any betamethasone given during pregnancy	566 (1.9)	111 (2.2)	68 (2.7)	0.0109
Betamethasone given during delivery hospitalization	200 (0.7)	38 (0.8)	11 (0.4)	0.3023
Gestational age at delivery, weeks	39.6 (38.9–40.4)	39.4 (38.7–40.3)	39.1 (38.3–39.7)	< 0.0001

Data presented as n (%) and median (interquartile range)

Bivariate analyses performed using chi-square tests for the categorical values and Kruskal-Wallis tests for continuous variables

In the overall cohort, the perinatal morbidity composite occurred in 8,601 (23.1%) of the infants. In unadjusted analyses (Table 3), infants born to patients in the intermediate screening group had a higher risk for the perinatal composite outcome compared to those born to patients in the normal screening group (OR 1.23, 95% CI 1.15–1.32). The risk was higher for infants born to patients in GDM group compared to those born to patients with normal screening (OR 1.86, 95% CI 1.70–2.20). After adjusting for potential confounders, these increased risks for the composite perinatal morbidity outcome persisted for those with intermediate screening (aOR 1.18, 95% CI 1.10–1.26) and those with GDM (aOR 1.58, 95% CI 1.44–1.74) compared to those with normal screening. Each of the individual components of the perinatal composite occurred significantly more frequently in patients with GDM compared to normal screening except for stillbirth. For those with intermediate screening compared to normal screening, the components that occurred more frequently were LGA (OR 1.52, 95% CI 1.39–1.66) and neonatal hypoglycemia requiring NICU admission (OR 1.29, 95% CI 1.13–1.48), but not NICU admission, respiratory distress, birth injury or stillbirth.

**Table 3:** Association between perinatal morbidity composite and glucose screening result grouping

	Normal screening (*n* = 29,698)	Intermediate screening (*n* = 5,092)	GDM (*n* = 2,487)	*P* value	Intermediate screen vs normal screen OR (95% CI)	GDM vs normal screen OR (95% CI)	Intermediate screen vs normal screen aOR (95% CI)^a^	GDM vs normal screen aOR (95% CI)
Composite perinatal morbidity outcome	6456 (21.7)	1299 (25.5)	846 (34.0)	<.0001	1.23 (1.15-1.32)	1.86 (1.70-2.03)	1.18 (1.10-1.26)	1.58 (1.44-1.74)
LGA^b^	2712 (9.1)	675 (13.3)	364 (14.7)	<.0001	1.52 (1.39-1.66)	1.71 (1.52-1.92)		
Hypoglycemia requiring NICU admission^c^	1223 (4.1)	267 (5.2)	316 (12.7)	<.0001	1.29 (1.13-1.48)	3.39 (2.97-3.86)		
NICU admission	3845 (13.0)	697 (13.7)	570 (22.9)	<.0001	1.07 (0.98-1.16)	1.99 (1.81-2.21)		
Respiratory distress^c^	1204 (4.1)	227 (4.5)	146 (5.9)	<.0001	1.10 (0.96-1.28)	1.48 (1.24-1.76)		
Birth injury^d^	148 (0.5)	30 (0.6)	22 (0.9)	0.035	1.18 (0.80-1.75)	1.78 (1.14-2.79)		
Stillbirth	34 (0.1)	4 (0.1)	5 (0.2)	0.335	0.69 (0.24-1.93)	1.76 (0.69-4.50)		

Data are presented as n (%) unless otherwise specified; the normal screening group is the referent group for all comparisons

Multivariable logistic regression models performed and adjusted for potential covariates using the maternal and fetal characteristics with p-values < 0.05 in bivariate analyses

Abbreviations: GDM = gestational diabetes mellitus; GCT = glucose challenge test; GTT = glucose tolerance test; OR = odds ratio; CI = confidence interval; aOR = adjusted odds ratio; LGA = large for gestational age; NICU = neonatal intensive care unit

^a^Adjusted for all baseline characteristics with p-value < 0.05 (parity, race/ethnicity, insurance type, body mass index, marital status, chronic hypertension, in vitro fertilization, gestational age at delivery, preconception metformin use, aspirin use, betamethasone use, and maternal age)

^b^LGA missing for 23 births

^c^Respiratory distress includes meconium aspiration syndrome, neonatal bronchopulmonary disorder, and other neonatal respiratory distress

^d^Birth injury includes neonatal brachial plexus injury, Erbs palsy, Klumpkes palsy, or fracture of the clavicle, femur, humerus, skull or other fracture

Among the secondary outcomes, patients with intermediate screening were significantly more likely to deliver by cesarean (OR 1.37, 95% CI 1.28–1.46), have HDP (OR 1.30, 95% CI 1.120–1.40), and significantly less likely to deliver SGA infants (OR 0.73, 95% CI 0.65–0.81) compared to patients with normal screening results (Table 4).

**Table 4:** Association between secondary outcomes and glucose screening result grouping

	Normal screening (*n* = 29,698)	Intermediate screening (*n* = 5,092)	GDM (*n* = 2,487)	*P* value	Intermediate screen vs normal screen OR (95% CI)	GDM vs normal screen OR (95% CI)
SGA^a^	3064 (10.3)	392 (7.7)	191 (7.7)	<.0001	0.73 (0.65-0.81)	0.72 (0.62-0.84)
Cesarean delivery	8254 (27.8)	1755 (34.5)	981 (39.5)	<.0001	1.37 (1.28-1.46)	1.69 (1.56-1.84)
OASIS	551 (1.9)	87 (1.7)	51 (2.1)	0.57	0.92 (0.73-1.16)	1.11 (0.83-1.48)
HDP	4442 (15.0)	945 (18.6)	538 (21.6)	<.0001	1.30 (1.20-1.40)	1.57 (1.42-1.74)

Data presented as n (%) unless otherwise specified; the normal screening group is the referent group for all comparisons

Multivariable logistic regression models performed

Abbreviations: GDM = gestational diabetes mellitus; OR = odds ratio; CI = confidence interval; SGA = small for gestational age; OASIS = obstetric anal sphincter injuries; HDP = hypertensive disorders of pregnancy

^a^Data missing for 23 neonates

## Discussion

Among patients who had a documented 1-hour GCT test at ≥ 30 24 weeks 0 days of gestation, 13.7% had an intermediate screening result and 6.6% had testing results consistent with a diagnosis of GDM. Patients in both testing categories experienced increased risks for adverse pregnancy outcomes including the perinatal morbidity composite compared to patients with normal screening results, suggesting that even those with mild glucose dysregulation in pregnancy remain at increased risk. Within the individual components of the perinatal morbidity composite, newborns born to patients with intermediate screening had higher frequencies of LGA and hypoglycemia requiring NICU admission compared to patients with normal screening. Both patients with GDM and intermediate screening experienced higher rates of cesarean delivery and HDP.

Our results compare similarly to prior studies in which abnormal glucose screening was associated with higher incidences of obstetric and neonatal complications compared to normal screening [13–18], with most showing an association between maternal glucose dysregulation and macrosomia or LGA infants. A 2016 systematic review and meta-analyses found that, when compared to individuals with zero elevated GTT values, those with one elevated GTT value had significantly worse pregnancy outcomes including higher rates of LGA infants, neonatal hypoglycemia, NICU admissions, respiratory distress syndrome, Apgar < 7 at five minutes, cesarean section, and HDP [14]. In a more recent systematic review and meta-analysis [13], patients with abnormal screening had higher rates of cesarean delivery, HDP, macrosomia, and shoulder dystocia compared to those with normal screening [13]. However, this study did not demonstrate differences in frequencies of NICU admissions, Apgar scores, or respiratory morbidity. While these authors used BMI, maternal age and GCT values as covariates in their analyses, there are other possible confounders which may contribute to the heightened risks observed.

In our study, we were able to adjust for important potential confounders in addition to maternal age and BMI, such as medical comorbidities, sociodemographic data and aspirin, metformin and steroid use. A prior study by Sermer et al. also established a graded increase in risk associated with maternal glucose intolerance among a Canadian cohort. These authors found that increasing glucose values on GTT testing is an independent predictor for various outcomes including macrosomia, cesarean section, HDP, and maternal or neonatal length of stay, with higher values increasing risk of cesarean and macrosomia after adjustment for parity, BMI, maternal age, and hypertension [19]. Similarly, Ju et al. previously found a similar relationship between those with abnormal GCT without GDM and elevated risk of perinatal outcomes compared to normal screening controls among a cohort of primiparous Australian patients; however when authors adjusted for maternal age and BMI, this relationship was no longer significant [16]. Unlike these prior studies, our population consists of patients from a diverse, urban area within the United States. Furthermore, the adjusted analyses in our study are comprehensive and demonstrate persistent associations between maternal hyperglycemia and neonatal risks despite differences in characteristics previously not controlled for such as maternal medical comorbidities, sociodemographic information, and delivery characteristics (including mode of delivery and gestational age). These results demonstrate an exposure-responsive relationship with comparative risks for specific outcomes between those with normal screening, intermediate screening, and GDM. Our findings suggest that maternal glucose dysregulation represents a spectrum of disease with increasing hyperglycemia linked to poorer outcomes, even in those without GDM.

We recognize that our frequency of GDM is low (6.6%) as opposed to the 8% incidence reported in the general population [1, 2]. However, our cohort was limited to singleton pregnancies with infants born > 35 weeks and we excluded patients with early diagnosis of GDM (< 168 days of gestation) as these patients are more comparable to those with pre-gestational diabetes and would likely confer additional bias to the data.

It is well-established that treatment of GDM reduces perinatal morbidity [5], however it remains unclear how treatment of those with mild glucose dysregulation may augment perinatal risk. Current practice does not dictate any interventions for patients with abnormal glucose screening without diabetes, however therapy may be beneficial. In one study, Langer et al. demonstrated that strict glycemic control among patients with one abnormal oral glucose tolerance test value lead to reductions in LGA infants and neonatal morbidity [20].

The mainstay of treatment for GDM is lifestyle interventions which includes a combination of diet, exercise, and glucose monitoring. Medication use is reserved for those with persistent elevations in glucose testing. For patients with GDM, lifestyle interventions alone have been associated with reductions in risk of LGA infants and neonatal fat mass; however, data remains inconclusive on reductions in composite neonatal outcomes, hypoglycemia, or perinatal death associated solely with diet and exercise interventions [21, 22]. Lifestyle interventions have also been examined as a possible prevention strategy for GDM among low risk patients with conflicting results [23–25]. While diet and exercise alone may not reduce incidence of GDM or significantly reduce perinatal outcomes alone for patients with GDM, perhaps there remains a role for such interventions for risk reduction in those with mild hyperglycemia.

In a Cochrane review of treatment for patients with abnormal GDM screening, dietary counseling and glucose monitoring were found to reduce the rate of LGA infants without changing rates of cesarean or operative vaginal delivery [26]. Gestational weight gain is another contributor to macrosomia [27]. Thus, it is possible that lifestyle changes to help prevent excessive gestational weight gain alone may help to reduce the risk of macrosomia. Further research is needed on whether interventions with diet, exercise, or glucose monitoring may be beneficial in reducing neonatal risks for these patients. Interestingly, an Australian cost analysis comparing interventions for treatment of mild gestational diabetes compared to routine prenatal care found that, for high-income countries, the possible reduction in perinatal morbidity and mortality would justify healthcare system costs of interventions [28].

We found that abnormal glucose screening is associated with a higher frequency of HDP, which is consistent with prior studies [29, 30]. Therefore, consideration should be given to increased counseling and observation for blood pressure elevations for those with glucose intolerance. Further, we found higher frequencies of LGA infants and cesarean delivery in both the intermediate screening and GDM groups, with no observed differences in the frequencies of operative delivery. As macrosomia is a known risk factor for labor dystocia, this may reflect labor arrest disorders, but we unfortunately did not have data on the indication for cesarean. It is also possible that obstetricians may have been less inclined to perform an operative vaginal delivery if they were concerned about macrosomia. In the most recent meta-analysis by Roeckner et al., patients with abnormal screening were found to have higher rates of shoulder dystocia (1.8% vs. 1.1%) compared to the control group with normal screening [13], although the incidences were still low. In our study, we included brachial plexus injuries and bony fractures as markers for severe shoulder dystocia in the definition of birth injury but did not find a difference.

More recently, it has been shown that up to 33% of patients with risk factors and normal GCT screening in the early third trimester will end up testing positive for GDM on GTT if re-tested later in gestation [31]. Moreover, post-prandial glucose values do not elevate significantly until mid-late pregnancy [32]. Within the current study, data on gestational age at time of glucose screening was not analyzed; thus, it remains possible that many of the patients classified in the intermediate group may ultimately have been diagnosed with GDM if repeat GTT was performed at a later date. The American College of Obstetrics and Gynecology currently gives a window of 24–28 weeks’ gestation for GDM testing. Given the increased risk for those with intermediate screening and the potential that many of those patients may later be diagnosed with GDM if re-tested, investigation into the appropriate timing of GDM screening for patients with risk factors is warranted. Interestingly, newer evidence suggests that alternative modalities for GDM screening, such as continuous glucose monitoring (CGM), may also provide improved accuracy in diagnosis and allow for more tailored treatment to further reduce perinatal complications [33–35]. Using CGMs for GDM screening may represent not only a more patient-centered approach but also more specific data for diagnosis, particularly for patients with risk factors for abnormal GDM screening.

Lastly, while it is known that gestational diabetes increases maternal risk of developing diabetes (predominantly type 2 diabetes) [36], heart failure [37], cardiovascular disease [38], and liver disease [39], little evidence exists on the long-term maternal risks associated with abnormal glucose screening. Retnakaran et al. found that elevated post-load values on GTT are more predictive of postpartum diabetes [40], however further studies to better understand risk stratification in this population based on GTT values may prove useful in motivating patients to pursue healthy life choices or follow-up care with primary care providers. This study has several strengths. First, it examines a large sample that is racially, ethnically, and socioeconomically diverse. We minimized confounding by adjusting for covariates that are known risk factors for GDM as well as for the primary and secondary outcomes. Additionally, our data are collected from a reliable EMR system. Individual samples of each screening category were reviewed and verified to confirm data extraction was correctly performed.

However, this study is not without limitations. First, this is a single-center, retrospective study using a large EMR dataset. While the large sample size provides statistical power, detailed chart review of specific variables was not possible. For example, only patients with both GCT and GTT testing were included in this cohort. Therefore, individuals who were unable to complete the GTT or who alternatively chose glucose fingerstick monitoring for diagnosis were excluded. This may introduce selection bias as the majority of those patients who are high risk for GDM would likely be strongly encouraged to complete the GTT. Additionally, this may miss patients with abnormal GCT testing who opted to be treated as GDM. Our data also did not include information regarding fetal anomalies, so no exclusions of this type could be applied which could also influence the primary composite outcome. Among the groups with missing data, a select sample of patients was identified for individual record review to confirm missing data and proper classification of individuals within the cohort. Second, our eligibility criteria may limit generalizability of our results to excluded populations, including those with differing baseline rates of GDM and multifetal pregnancies.

## Conclusions

This study highlights that patients with abnormal glucose screening are still at increased risk for adverse pregnancy outcomes compared to those with normal screening, including neonatal risks of hypoglycemia and LGA and maternal risks of cesarean delivery and HDP. Further research is needed to understand if further counseling or dietary interventions may benefit this population.

## Electronic supplementary material

Below is the link to the electronic supplementary material.

Supplementary Material 1

## Data Availability

The datasets used and/or analyzed during the current study are available from the corresponding author upon reasonable request.

## Abbreviations

AOR: Adjusted Odds Ratio
BMI: Body Mass Index
CI: Confidence Interval
EDD: Estimated Due Date
EMR: Electronic Medical Record
GCT: Glucose Challenge Test
GDM: Gestational Diabetes Mellitus
GTT: Glucose Tolerance Test
HDP: Hypertensive Disorders of Pregnancy
ICD: International Classification of Diseases
IVF: In Vitro Fertility
LGA: Large For Gestational Age
NICU: Neonatal Intensive Care Unit
OASIS: Obstetric Anal Sphincter Injuries
OR: Odds Ratio
SGA: Small for Gestational Age
